# Aqueous Photochemistry of 2-Oxocarboxylic Acids: Evidence, Mechanisms, and Atmospheric Impact

**DOI:** 10.3390/molecules26175278

**Published:** 2021-08-31

**Authors:** Marcelo I. Guzman, Alexis J. Eugene

**Affiliations:** Department of Chemistry, University of Kentucky, Lexington, KY 40506, USA; alexis.eugene@uky.edu

**Keywords:** pyruvic acid, glyoxylic acid, cross-photoreaction, dissolved O_2_, photolysis, SOA, air-water interface, quantum yield, cloud, fog

## Abstract

Atmospheric organic aerosols play a major role in climate, demanding a better understanding of their formation mechanisms by contributing multiphase chemical reactions with the participation of water. The sunlight driven aqueous photochemistry of small 2-oxocarboxylic acids is a potential major source of organic aerosol, which prompted the investigations into the mechanisms of glyoxylic acid and pyruvic acid photochemistry reviewed here. While 2-oxocarboxylic acids can be contained or directly created in the particles, the majorities of these abundant and available molecules are in the gas phase and must first undergo the surface uptake process to react in, and on the surface, of aqueous particles. Thus, the work also reviews the acid-base reaction that occurs when gaseous pyruvic acid meets the interface of aqueous microdroplets, which is contrasted with the same process for acetic acid. This work classifies relevant information needed to understand the photochemistry of aqueous pyruvic acid and glyoxylic acid and motivates future studies based on reports that use novel strategies and methodologies to advance this field.

## 1. Introduction

The Earth’s atmosphere is not just made up of gaseous chemicals but also contains very small particles known as aerosols. Atmospheric aerosols, suspensions of fine solid, semisolid, or liquid particles in air (e.g., smog, haze, smoke, fog, and mist) can have very large and important effects on the environment. A well-known example of these environmental effects is smog formation in large cities, where the particle concentration has become too high. Breathing in the smog caused by these particles can aggravate respiratory problems like asthma, and even be correlated to causing cancer in the long term. The particles also have major effects on the Earth’s climate by directly preventing sunlight from reaching the ground or by forming clouds that block incoming sunlight.

Much like how meteorologists can predict tomorrow’s weather, climate scientists can predict what effects the particles will have on Earth’s future climate and on the health of humans. This work can contribute to improving these predictions from new knowledge and understanding of chemical reactions that occur within the particles and on their surface. Specifically, the work covered in this review studies what happens to two chemicals commonly found in water based atmospheric particles, specifically glyoxylic acid and pyruvic acid, when they are exposed to sunlight. The discoveries presented below show that both chemicals undergo complex reaction steps in water under a variety of conditions to form products that can contribute to an increase in the number of atmospheric particles on Earth. The quantum efficiency measured for each reaction is available in the literature discussed below to be used by climate scientists in models with improved predictive accuracy. The improved understanding provided by the work reviewed below should help to protect the health of humans by preventing harmful pollution events. The work should also contribute toward guiding decisions about environmental policy.

### 1.1. Defining the Importance of Studying Small 2-Oxocarboxylic Acids

The aqueous photochemistry of small 2-oxocarboxylic acids is a topic that has garnered much attention in the literature recently [1,2,3,4,5,6,7] because of the complex mechanisms of reaction involved for these ubiquitous chemical species. The chemical behavior of these small molecules is of interest to a wide variety of disciplines because they are common waypoints in the chain of oxidation of various organic molecules, for example the atmospheric processing of aromatic emissions [8,9,10,11,12], of both biogenic and anthropogenic origin. 2-Oxocarboxylic acids are also key species in the chain of reactions during glycolysis [13] and the reductive tricarboxylic acid cycle [14]. The prevalence of small 2-oxocarboxylic acids across many different chemical environments, from atmospheric water to the inside of cells, demands a thorough understanding of their transformation pathways. This review explores the behavior and photochemical transformations of small 2-oxocarboxylic acids in the context of atmospheric aqueous chemistry.

### 1.2. The Atmospheric Chemistry and Secondary Organic Aerosol Framework

The Earth’s atmosphere is a dynamic, chemically rich environment made up of organic and inorganic species partitioned between the gas phase and solid, semisolid, and liquid particles suspended in the air. These particles, or aerosols, vary widely in size (~1 nm to 20 μm) [15] and chemical composition. Aerosols are typically divided into size ranges by their diameters, resulting in two broad classifications: fine aerosol (typically particles smaller than 1 μm), and coarse aerosol (particles larger than 1 μm) [15]. Coarse aerosol is primarily composed of inorganic particles like dust or sand that are mechanically dispersed into the air. Fine aerosol is composed of a mixture of inorganics and organic compounds, the ratio of which varies based on the source of the aerosol. The inorganic species present in fine aerosol are primarily ammonium and sulfate ions with some variable content of nitrate. Fine aerosol also contains liquid water because of the high hygroscopicity of its other components. The organic component of fine aerosol is made up of many different water soluble species containing alcohols, carbonyls, oxidized aromatic compounds, and organic acids including 2-oxocarboxylic acids [15,16]. The mass fraction of organics in fine aerosol can be anywhere from 20–90% depending on the source of the aerosol reported [17,18,19]. The most abundant 2-oxocarboxylic acids in the atmosphere, and therefore most likely to contribute a significant impact on atmospheric chemistry, are glyoxylic acid (GA, a two carbon molecule) and pyruvic acid (PA, a three carbon molecule) [16,20].

GA and PA are semivolatile species that exist in both the gas phase and the particle phase. However, for both species, most of the carbonyl group gets hydrated forming a gem-diol. For GA, the *K*_hyd_ for this process is 300 at 25 °C [21] so ~99% of GA exists as the gem-diol, while only 68% of PA is hydrated (*K*_hyd_ = 2.10 at 25 °C) [22]. The existence of the less volatile gem-diol form enhances partitioning of GA and PA to the particle phase, where the majority is found according to their Henry’s law constants, *K*_H,GA_ = 1.1 × 10^2^ mol m^−3^ Pa^−1^ and *K*_H,PA_ = 3.1 × 10^3^ mol m^−3^ Pa^−1^ [23]. Average particle phase concentrations of GA have been observed to be 44.3 ng m^−3^ in urban aerosol [16] and 4.91 ng m^−3^ in pristine marine aerosol [20]. In the same measurements, PA was found at levels of 49.7 ng m^−3^ (urban environment) [16] and 0.79 ng m^−3^ (pristine environment) [20]. Similar ranges of GA and PA concentrations have been measured in various studies [24,25,26]. For the range of relative humidity 50–90% [27], the water content in aerosols is largely dominated by the deliquescence of ammonium bisulfate. This deliquescence requires that the liquid particles have a ratio of ~0.6 g H_2_O/1 g sulfate [28,29]. Simultaneous measurements of GA, PA, and sulfate predict aqueous concentrations of up to ~250 mM for both GA and PA in acidic urban aerosols.

**Figure 1:**
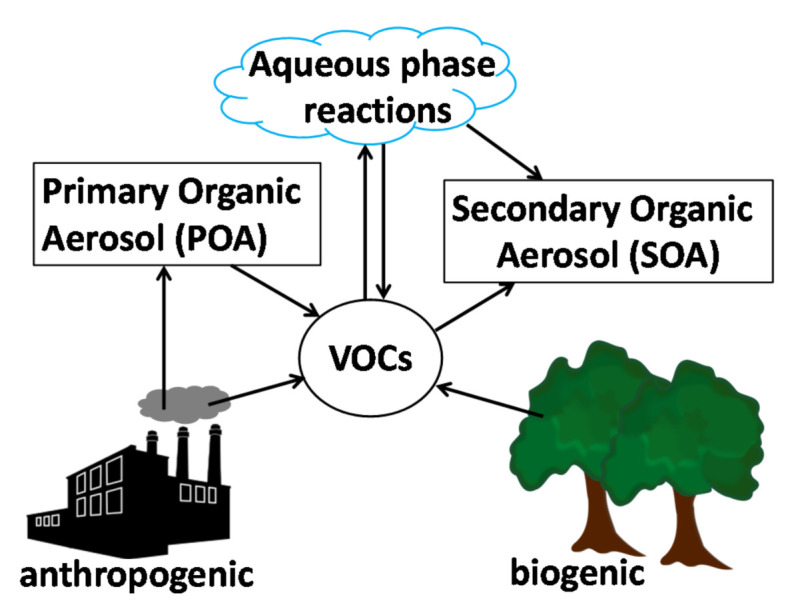
Pictorial representation showing formation pathways of secondary organic aerosol (SOA) species in the atmosphere from volatile organic compounds (VOCs).

Figure 1 shows a general formation scheme of the different components of organic aerosol. Organic aerosol is further divided based on the originating formation mechanism into primary organic aerosol (POA) and secondary organic aerosol (SOA). POA is composed of solids or liquids emitted directly to the atmosphere from anthropogenic sources like combustion engines and cooking fires, or from natural biogenic sources, as shown in Figure 1. Traditionally, SOA has been thought of as being formed from the gas-to-particle condensation of lower volatility products from the oxidation of volatile organic compounds (VOCs) or by the aging (oxidation) of POA [17,30]. Thus, most of the literature in the past has focused on these pathways [31]. However, much recent work has begun to recognize the importance of aqueous chemistry occurring in cloud and fog droplets to the formation of SOA [31,32,33,34,35]. Within the framework of Figure 1, GA and PA are both formed during the oxidation of aromatic species emitted as POA and organic gases from combustion [11,36,37] and biomass burning [8,9,10,38]. They are also formed during the processing of the isoprene given off by plants [39,40,41,42], which is the most abundant nonmethane VOC emitted to the atmosphere each year [43]. It is estimated that 15.72 Tg of glyoxylic acid and 0.85 Tg of pyruvic acid are produced in the atmosphere every year, primarily in the aqueous phase [44].

The broad range of sources and chemical compositions of the aerosols discussed above immensely alters the effect aerosols have on the Earth’s energy balance, which is the balance between incoming and outgoing radiation. In the absence of other influences, more incoming than outgoing radiation produces a warming effect on the Earth’s surface and vice versa. Aerosol effects on this energy balance fall into two categories. The first is through aerosol–radiation interactions, where incoming solar radiation is either absorbed or scattered by the aerosol, depending on the composition and optical properties of the aerosol [45]. Aerosol that absorbs radiation contributes a net warming effect to the Earth’s atmosphere, while aerosol that scatters radiation reduces the amount of radiation that enters the atmosphere, providing a net cooling effect. The second way aerosol affects the energy balance is through the aerosol–cloud interaction. Aerosols act as cloud condensation nuclei, providing seeds where larger cloud droplets can grow and enhancing cloud cover of the Earth, thereby reflecting more solar radiation and causing a net cooling effect on the environment [45]. Aerosol effects are directly related to the mass loading of particles in the air, dictating that any quantification of these effects needs the most accurate accounting possible of how much SOA is present under various conditions.

**Figure 2:**
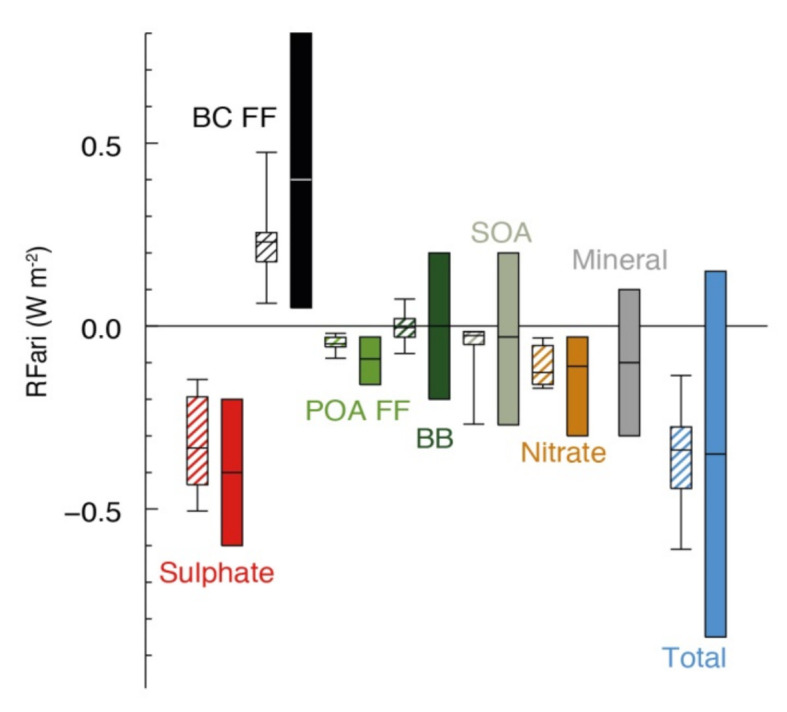
Annual mean top of the atmosphere radiative forcing due to aerosol–radiation interactions (RFari, in W m^–2^) due to different anthropogenic aerosol types, for the 1750–2010 period. Hatched whisker boxes show median (line), 5th to 95th percentile ranges (box) and min/max values (whiskers) from AeroCom II models [46] corrected for the 1750–2010 period. Solid colored boxes show the AR5 best estimates and 90% uncertainty ranges. BC FF is for black carbon from fossil fuel and biofuel, POA FF is for primary organic aerosol from fossil fuel and biofuel, BB is for biomass burning aerosols, and SOA is for secondary organic aerosols—reproduced with permission from ref. [45].

The extent to which aerosols contribute to cooling or warming is well understood for many types of aerosols, but the contribution from SOA is much less certain. Figure 2 shows the estimated effect of aerosols on the atmosphere presented as the radiative forcing due to aerosol–radiation interactions (RFari) with uncertainties and for various types of aerosol. The estimates in Figure 2 were synthesized from a large body of peer reviewed literature by the Intergovernmental Panel on Climate Change (IPCC) in their fifth assessment report [45]. Formally, the radiative forcing in Figure 2 is the change in the total downward flux of incoming radiation at the top of the troposphere due to the presence of aerosol particles directly interacting with radiation. This means that a negative RFari denotes a reduction of radiation reaching Earth’s surface and a net cooling effect caused by that type of aerosol. One can observe from Figure 2 that the uncertainty in the RFari for SOA is very large compared to the magnitude for both modeling methods. It is thought that this uncertainty is due to atmospheric models predicting SOA loadings much lower than what is observed by field studies, and that one of the main reasons for the underprediction is that there are other mechanisms of SOA formation not yet incorporated into the models [18]. In order to improve the models, these unknown mechanisms need to be identified, have their significance to the real atmosphere evaluated, and have their physical parameters such as rate constants and quantum yields measured in the laboratory. The aqueous photochemistry of 2-oxocarboxylic acids is likely one of these missing sources that needs to be characterized and included in the models.

## 2. Atmospheric Photochemistry and Small 2-Oxocarbxylic Acids

Sunlight is overwhelmingly the largest source of energy input on the Earth [47]. As such, it is the driving force for most of the chemical transformations that occur in the atmospheric gas and aqueous phases [34]. The majority of organic aerosol can be found in the troposphere where sunlight of wavelengths greater than 290 nm penetrates [48]. The troposphere is the lowest layer of the atmosphere that extends from the Earth’s surface to about 10–16 km depending on the current temperature, underlying surface configuration, latitude, and time of day [49]. The primary reason for the 290 nm cut off in the troposphere is that ozone (O_3_) in the layer directly above the troposphere, called the stratosphere, filters out much of the light between 220 and 330 nm [49]. The total solar irradiance that reaches the Earth’s atmosphere is on the order of 1360 W m^−2^ [47], a huge amount of energy that allows many varied light-dependent reactions to occur.

In atmospheric chemistry, organic molecules typically undergo two types of photochemical reactions: direct and indirect photolysis. Direct photolysis may occur if the species in question can absorb photons of the wavelengths available in sunlight. Typically, the importance of direct photolysis is evaluated by comparing quantum yields (Φ), which is defined as a number of events per photon absorbed [50]. For this work, Φ would be the number of molecules of reactant destroyed or of product generated per photon absorbed. Φ for many processes in the gas phase and in organic solvents have been reported in the literature, but aqueous photochemical processes have received little attention until recently, leading to a lack of Φ values for atmospheric models to evaluate the relative importance of each photochemical process [35]. If the molecule cannot absorb sunlight, indirect photooxidation can occur through reaction with photochemically produced hydroxyl radicals (HO^•^), which is the most important atmospheric oxidant in both the gas phase and the aqueous phase. For most organics, reaction with HO^•^ controls their primary fate due to absorption bands that are below 290 nm, or to the small molar absorption coefficients that make the typically fast HO^•^ reaction outcompete direct photolysis [35].

**Figure 3:**
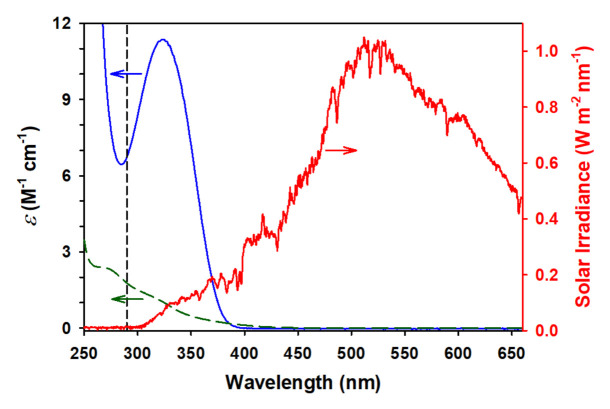
The molar absorption coefficients (ε, **left axis**) in the UV-visible absorption spectra of (**dashed green**) glyoxylic acid and (**solid blue**) pyruvic acid in water, and (**red** and **right axis**) direct solar irradiance spectrum from a sunny and cloudless day (19 August 2018) in Lexington, KY (USA) at 230 m above ground level. The vertical dashed line indicates the ozone cut-off at a wavelength λ_cut-off_ ≥ 290 nm.

In this regard, small 2-oxocarboxylic acids are a special case. Because of the conjugation of the two adjacent carbonyls, the n→π* transition of 2-oxocarboxylic acids’ absorption profiles are shifted to low enough energy that they can absorb sunlight directly, leading to complex radical chemistry. Figure 3 shows the UV-visible absorption spectra of aqueous GA (dashed green trace) and PA (solid blue trace) overlapped with the direct solar irradiance spectrum (red trace) recorded at noon on a sunny cloudless day at 230 m above ground level on 19 August 2018 in Lexington, KY, USA (elevation of 298 m above the see level). The vertical dashed line in Figure 3 indicates the minimum wavelength of solar radiation that can penetrate to ground level, 290 nm. The overlap between the spectra of GA and PA with the solar spectrum indicates that both 2-oxocarboxylic acids should be readily photoactivated to possibly form higher complexity, more oxidized products, contributing to SOA production in the atmosphere. Furthermore, two studies based on a combination of rate constants and absorption profiles have predicted that direct aqueous photochemistry should be a significant fate for PA, and may even be the dominant fate for PA rather than gas phase photolysis or gas/aqueous reaction with HO^•^ [1,35]. The removal by HO^•^ of GA and PA is relatively fast in the aqueous phase as they proceed with rate constants $k\;\approx \;10^{8}\;M^{-1}\;s^{-1}$ [51,52], resulting in average lifetimes of five days for both. Instead, the photochemical lifetime of GA and PA in atmospheric water are 11 days and 22 min, respectively [2,3]. Thus, GA can also be expected to undergo direct photolysis, albeit the slower rate. However, with the higher atmospheric production of GA than PA, its direct photolysis, while not the primary sink, has a significant impact on atmospheric composition, making both GA and PA contributors to SOA production.

Thus, given their potential importance to SOA production, this review encompasses work to identify the products of photoreactions and propose mechanisms for the aqueous photolysis of GA and PA under atmospherically relevant conditions. The quantum yields recently available in the literature are reported to incorporate these processes into atmospheric models of SOA production.

### 2.1. Background on the Photochemistry of Glyoxylic Acid in the Gas Phase

**Scheme 1:**
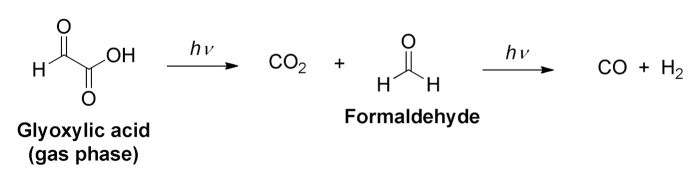
The main products of the gas-phase photochemistry of glyoxylic acid (GA) reported by Back and Yamamoto [53].

Before the most recent work covered here was performed, only one study of pure glyoxylic acid photochemistry was available in the literature. Back and Yamamoto conducted this study in the gas phase [53] at irradiation wavelengths of 239, 275, 346, 366, and 382 nm. They identified the main reaction products as CO2 and formaldehyde, with secondary CO and H_2_ production occurring from the photodecomposition of the produced formaldehyde as shown in Scheme 1. In addition, they were also able to distinguish a second source of CO independent from the formaldehyde. Although they were unable to identify formic acid, they postulated its presence as a coproduct when CO is formed. Theoretical work for the wavelength interval 350–380 nm supported the major gas-phase photolysis pathways correspond to photodecarboxylation by direct C-C bond cleavage (Norrish Type I reaction), with additional contribution form a channel for β-hydrogen transfer followed by CO loss [54].

**Scheme 2:**
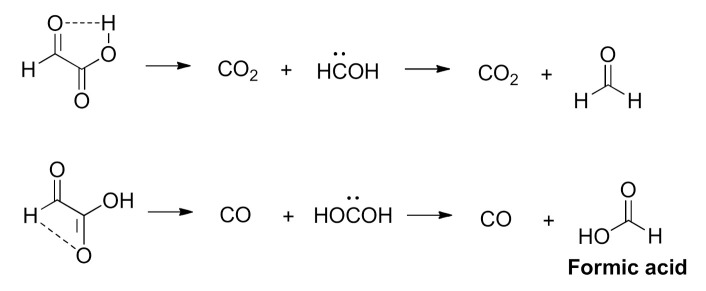
Mechanisms of intramolecular hydrogen atom transfer followed by dissociation during the gas-phase photochemistry of GA proposed by Back and Yamamoto [53].

The primary mechanistic pathways proposed to explain the products were all internal H atom transfer processes followed by dissociation to give the following: CO_2_/formaldehyde; CO/formic acid; and CO_2_/CO/H_2_, as shown in Scheme 2. Quantum yields were reported relative to the CO_2_/formaldehyde pair as 3% for CO/formic acid and 16% for CO_2_/CO/H_2_ at 275 nm [53].

**Scheme 3:**
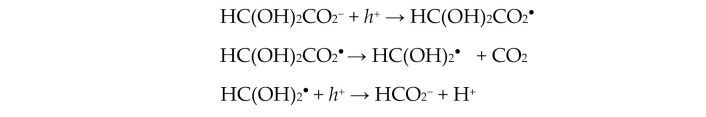
Photocatalytic oxidation of adsorbed glyoxylate on TiO_2_ [55].

In the 400 nm irradiation of gas phase GA adsorbed on TiO_2_, GA was photocatalytically converted to CO_2_, carbonate and formate [55]. Hydrated glyoxylate underwent a one electron loss to be oxidized forming an acyloxyl radical (HC(OH)_2_CO_2_^•^), which then decarboxylated to give CO_2_ and HC(OH)_2_^•^, which underwent an additional one electron loss oxidation coupled with deprotonation to make formate as shown below in Scheme 3:

Finally, the gas phase photochemistry of glyoxylate when embedded in cationic sodium chloride clusters has been recently described [56]. When the clusters were excited by 310–380 nm light, mass spectrometry was able to show that homolytic cleavage caused CO and HCO^•^ to be eliminated from glyoxylate, leaving behind formate and carbon dioxide radical anion (CO_2_^−^^•^) adducts with sodium chloride. The authors postulated that this type of reaction likely occurs in the troposphere.

### 2.2. Photochemistry of Glyoxylic Acid in Water

**Scheme 4:**
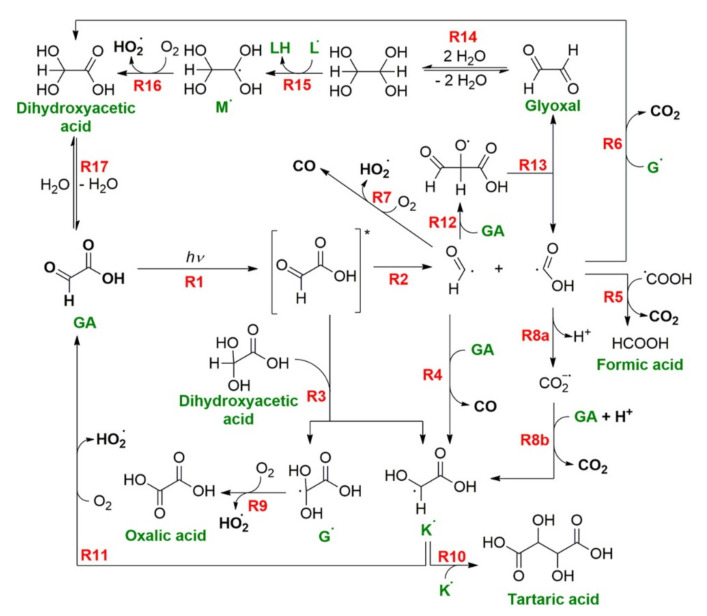
Detailed mechanism for the photooxidation of GA at λ ≥ 305 nm in Water, reproduced with permission from ref. [3].

The first study of the aqueous phase mechanism of GA direct photochemistry (λ ≥ 305 nm) in Scheme 4 is based on our previous work with 250 mM GA solutions at pH 1, which were studied doped (or undoped) with sodium chloride and sodium sulfate in amounts that mimic sea spray aerosols [3]. Surprisingly, irradiated aqueous GA formed many of the same observed or proposed products in the gas phase chemistry, including CO_2_, CO, and formic acid indicating similar reactive pathways are available in the gas and aqueous phases. However, glyoxal, oxalic acid, and tartaric acid were also formed in solution [3]. Overall, oxalic and tartaric acid formation was explained by a proton coupled electron transfer or hydrogen abstraction mechanism (indistinguishable), while the other products were formed through competing homolytic cleavage of the C-C bond [3]. Of particular interest was the formation of the most abundant product, glyoxal, because it is thought to be a major driver of SOA formation [57,58,59,60]. The quantum yield of GA loss to direct aqueous photolysis was reported to be 0.17%, which corresponds to an atmospheric lifetime of about 11 days, while the lifetime against indirect aqueous photolysis with HO^•^ was about five days, indicating that, whereas oxidation by HO^•^ is still the primary fate, direct photolysis of GA in water is still a competitive pathway.

**Scheme 5:**
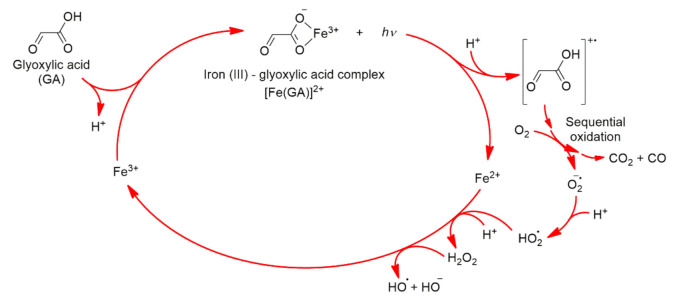
Mechanism for the iron (III) catalyzed photodecomposition of GA in water [63].

While the only study of the direct photochemistry of GA in water was described above [3], there are a few other relevant studies that arrive to related conclusions when investigating the effect of Fe^3+^ during irradiation. For example, Scheme 5 indicates that, upon 313 or 355 nm irradiation of complexes of iron (III) and GA in water, electron transfer proceeds from glyoxylate to the iron center [61,62], resulting in iron (II) and a radical cation [63], which based on our work contributes decarbonylation and decarboxylation reactions through formyl-oxyl radical (HOOC^•^) and formyl radical (^•^C(H)═O) intermediates [3]. The formyl radical can be further converted to formic acid by another iron (III) glyoxylate complex [61]. In the presence of dissolved O_2_, superoxide ion (O_2_^−^^•^), and its conjugated acid, the hydroperoxyl radical HO_2_^•^, (p*K_a_* = 4.8) is generated in the recycling of Fe^2+^ to Fe^3+^ [63]. These processes involving GA in the presence of iron (III) relevant to cloud water seem to favor the direct cleavage of the C-C bond. In addition, the formation of H_2_O_2_ depicted in Scheme 5 can also lead to further oxidation of organics by the Fenton generated HO^•^ in the presence of Fe^2+^.

### 2.3. Background on the Photochemistry of Pyruvic Acid in the Gas Phase

In contrast to GA, the photochemistry of PA has received far more attention, though key work is still a focus of interest, especially in the context of atmospheric reactions. In the gas phase, the early study of Vesley and Leermakers was able to detect CO_2_ and acetaldehyde in the 366 nm photolysis of PA in the gas phase [64]. Recently, the importance of this reaction generating acetaldehyde (and peroxyl radicals) was demonstrated to exceed ethane and propane oxidation by factors of ∼10 and ∼20 in the boreal environment [65]. A quantum yield for the decomposition of PA of 1.02 was reported [64], which was within experimental error of the value of 0.9 established decades later by Yamamoto and Back [66]. Both sets of authors contended that the primary photochemical decomposition pathway proceeds via an internal hydrogen atom transfer utilizing a cyclic transition state and methylhydroxycarbene as the intermediate yielding acetaldehyde [64,66]. The singlet methylhydroxycarbene was characterized upon 308 and 351 nm excitation to induce the photodissociation of gaseous PA [67,68]. Yamamoto and Back also implied that there were other nonvolatile products for them to identify using their gas chromatography (GC) method. Subsequently, Berges and Warneck were able to identify acetic acid and small amounts of CO and methane [69] for the same gas phase photolysis of PA. It was also proposed that an unknown pathway could result in the production of HO radical from the gas photolysis of pyruvic acid [70].

More recently, Reed Harris et al. were also able to detect methanol and formic acid among the gas phase products [71]. The quantum yield of PA decay was 0.24 ± 0.05 for ~100 ppm of PA in 600 torr of air when a small reactor was irradiated (λ = 290–380 nm) with a Xenon lamp filtered by a Pyrex window [71]. However, Reed Harris et al. later reported an updated quantum yield of Φ = 3.2 ± 0.5 for the photodecomposition of 3 ppm of gas phase PA irradiated at λ ≥ 300 nm inside a 4.2 m^3^ chamber at atmospheric pressure in dry air [72]. This significantly larger quantum yield was attributed to the much lower PA concentration used than in the earlier studies, as well as an enhancement due to reactions with O_2_ that were not observed before even though both studies were performed in air that is 21% O_2_ by volume, hence significantly more concentrated than PA in the reactors. An infrared spectroscopy study supported by density functional theory and ab initio calculations showed that the more stable PA conformer with an intramolecular hydrogen bond dominates the gas-phase photolysis (λ ≥ 290 nm) of pyruvic acid [73]. A water vapor molecule can disrupt the intramolecular hydrogen bond and decrease the photolysis rate constants [73].

### 2.4. Photochemistry of Pyruvic Acid in Water

In opposition to the gas phase photolysis of PA, a diversity of products and reaction mechanisms for the aqueous photochemistry of PA exists. Leermakers and Vesley contributed the first study of the aqueous photochemistry of pyruvic acid in 1963, using 285 mM solutions at natural pH, which were irradiated at λ ≥ 280 nm at 25 °C [74]. The same reaction was carried out in a variety of organic solvents including methanol, benzene, and chloroform for comparison. The authors supposed by analogy to the gas-phase reaction that in water a methylhydroxycarbene was likely a key reaction intermediate, and the primary products were CO_2_ in the headspace over the reaction and acetoin (H_3_CC(O)C(OH)CH_3_) in the liquid phase (from recombination of two methylhydroxycarbenes), which was identified by GC at 90 °C after removing the water and redissolving in methanol [74]. In all organic solvents except benzene, they reported that the main product was 2,3-dimethyltartaric acid (DMTA), identified by infrared (IR) and nuclear magnetic resonance (NMR) spectroscopies. Because the reaction did not proceed in benzene, the purported explanation provided was that, while the other organic solvents are protic, benzene is not, indicating a key role of the solvent in the solution phase mechanism. An additional experiment in water using sodium pyruvate instead of the acid yielded decomposition “…at least an order of magnitude slower,” but the products of this reaction were not characterized. A quantum yield of 0.79 was reported for the aqueous photoreaction of pyruvic acid, which was “more than 20 times larger” than for the conjugate base. Finally, Leermakers and Vesley speculated that the triplet excited state of pyruvic acid was the reactive species responsible for this chemistry based on the very large phosphorescence they observed upon irradiation (unpublished). Later, Kendall and Leermakers performed quenching experiments that further indicated that triplet excited state PA was the reactive species [75].

**Scheme 6:**
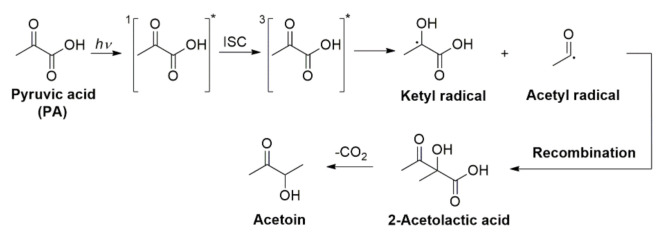
Early proposal by Closs and Miller of a multistep mechanism for pyruvic acid (PA) aqueous photolysis based exclusively on their interpretation of CIDNP data [76].

A decade later than Leermakers and Vesley, Closs and Miller undertook a study by chemically induced nuclear polarization (CIDNP) as the unique technique to explore the mechanism of PA photoreaction in water [76]. CIDNP is a nuclear magnetic resonance (NMR) technique used to study radical processes wherein the signals of triplet radical pairs can be observed. In these experiments, the NMR spectra of the 100 mM solutions of PA were able to be recorded during in situ irradiation at λ ≥ 310 nm. Closs and Miller proposed that, in both hydrogen donating solvents and nonreducing solvents, i.e., water, the mechanism involved free radical recombinations due to the observation of steady state signals only during irradiation, which disappeared after the light was shuttered. Using the chemical shifts of the radical pairs in this single technique, they tentatively assigned the identities of the radicals present in water. The formation of acetyl radical and PA ketyl radical (also called lactic acid radical) via H atom transfer from an –OH (which is actually unlikely) was proposed by Closs and Miller to proceed in multiple steps. These last two radicals finally recombine into 2-acetolactic acid (Scheme 6) that would decompose into acetoin. However, Closs and Miller did not include any further structural verification of these species and, at variance of the summarized representation in Scheme 6, the radicals for the pair needed to form 2-acetolactic acid would not form contiguously in solution but in separate photochemical cages [77].

Additional experiments with added naphthalene, a known triplet quencher, again indicated that the triplet state was involved in the observations of Closs and Miller. The authors postulated that the acetyl and ketyl radicals simply recombine to form 2-hydroxy-2-methylacetoacetic acid (also called 2-acetolactic acid), expected to account for ~30% of the organic products in their solutions [76]. Finally, Closs and Miller described their disappointment for the failed attempt to synthesize 2-acetolactic acid because it decarboxylated into acetoin too quickly to record the NMR spectrum of the parent 2-acetolactic acid, begging the question, if the primary photoproduct really was 2-acetolactic acid, why did it not also hydrolyze immediately into acetoin? These results also bring in to question whether the acetoin inconclusively observed earlier by Leermakers and Vesley [74] could also be a decomposition product of the true photochemical product since they only identified it after subjecting the sample to GC at 90 °C.

**Scheme 7:**
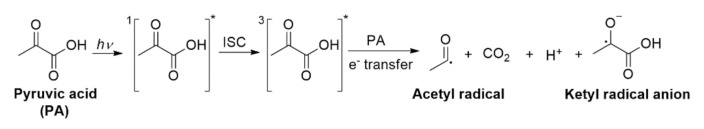
Initial step of PA(aq) photolysis by Davidson et al. [78,79].

A few years after the study by Closs and Miller, Davidson et al. were able to determine that the triplet excited state yield of the 350 nm PA photolysis in water was 0.22 with a lifetime of 74 ns [78]. Davidson et al. also theorized that, since the earlier studies showed that quantum yields were dependent on the type of solvent used during the photolysis, homolytic cleavage was unlikely to be the mode of reaction that forms the ketyl and acetyl radicals [78]. Instead, for the 350 nm photolysis, they proposed that bimolecular electron transfer occurred between the excited state of PA and a ground state PA molecule, shown in Scheme 7. The solvent influence on the reaction was then explained by highly polar solvents stabilizing the radical intermediates. However, they explained that this reaction should be encouraged by the presence of pyruvate anion, which is at odds with the documented suppression of photoreactivity in solutions of sodium pyruvate [74]. In a related publication, the same authors proved the presence of electron transfer during the reaction by demonstrating an enhancement of the yield of CO_2_ when electron acceptors were added to the solution [79].

**Scheme 8:**
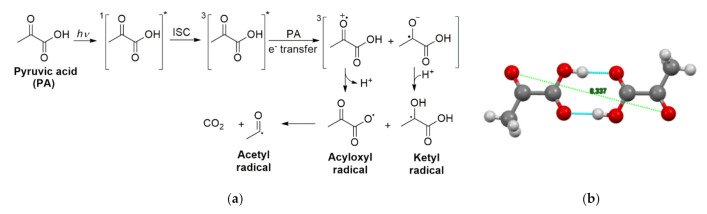
(**a**) Proposed mechanism of proton-coupled electron transfer in the 320 nm photochemistry of aqueous PA by Guzman et al. [80]; (**b**) crystal structure of the dimer of pyruvic acid with (light blue) intermolecular hydrogen bonds, which indicates a (green) distance of 8.337 Å for the oxygen atoms in the carbonyl centers of the crystal structure [81].

After a long hiatus, research on the photochemical mechanism of PA in water was revived by Guzman et al., who were able to trap the radicals produced during λ = 320 nm ± 10 nm photolysis of pyruvic acid by irradiating in frozen (77 K) aqueous solutions [80]. They were then able to use electron paramagnetic resonance spectroscopy to directly observe the organic radicals, which showed that the ketyl and acetyl radicals existed as triplet radical pairs (rather than as individual isolated radicals) with each unpaired spin separated by ≥0.5 nm, which could not be explained by a reaction pathway involving homolytic cleavage. These radical pairs remained completely stable when cycling their temperatures between 10 and 160 K but disappear irreversibly upon warming above ~190 K (the onset of ice diffusivity). However, the release of CO_2_ under cryogenic conditions was observed above the sublimation temperature of CO_2_ (140 K), which demonstrated that the ultrafast decarboxylation of a primary radical must occur while the radical pairs were stable. Thus, CO_2_ must have been produced by the fast and efficient decarboxylation of a primary acyloxyl radical generated immediately following photon absorption and deprotonation, which resulted in the detectable acetyl radical observed as part of the triplet pairs [80]. The large separation of the unpaired spins was rationalized to result from long range electron transfer occurring between a photoexcited PA and a ground state PA molecule (shown in Scheme 8a), coupled with a proton transfer to form a PA ketyl radical and an acyloxyl radical. As stated, the acyloxyl radical subsequently decarboxylated to yield primary CO_2_ and an acetyl radical. Because the actual separation of one of the radical pairs detected was ~8.7 Å, the data indicated that the unpaired electrons resided on the oxygen atoms in the carbonyl centers of two PA molecules forming a dimer [80], which are separated in the crystal structure by 8.3 Å as displayed in Scheme 8b [81].

**Scheme 9:**
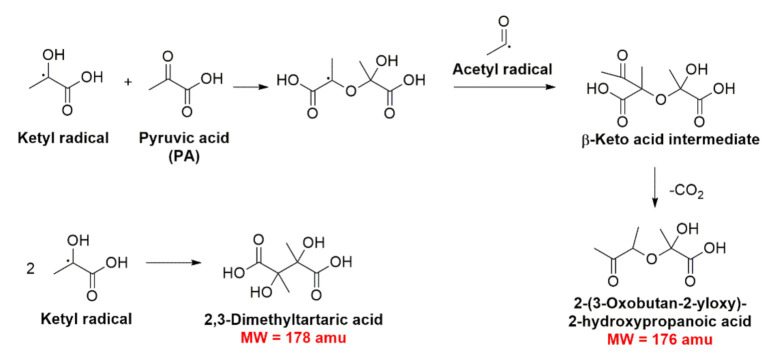
Formation of the organic products of PA photochemistry in water and ice from ketyl and acetyl radical intermediates identified by Guzman et al. [5,80,82].

Next, Guzman et al. characterized the organic products of the oxygen-free photoreaction at 313 nm of aqueous solutions adjusted at pH 1.0 both in the liquid state at 293 K [5] and in the frozen state between 227 and 269 K [82], using high pressure liquid chromatography with UV-visible and mass spectrometric (MS) detection for experiments with natural abundance and isotopic labelled PA [5]. In these studies in water and ice, two major organic products were identified in the condensed phase, while CO_2_(g) evolution kinetics was monitored in real time by Fourier transformed infrared (FTIR) spectroscopy. The first product was a six-carbon carboxylic acid with a neutral mass of 178 amu. The number of carbons was easily identified by using uniformly labeled ^13^C PA for the reaction and then analyzing by MS. The MS fragmentation spectrum of this species was consistent with a dicarboxylic acid. Additionally, the MS spectra from a reaction in H_2_O using PA deuterated only at the methyl group indicated that this six carbon dicarboxylic acid contained two methyl groups [5]. All together, the data for this product were consistent with the 2,3-dimethyltaratric acid previously proposed by Leermakers and Vesley [74]. However, the second organic product was more complex. Using the same methods, Guzman et al. were able to determine that the second product was a carboxylic acid of mass 176 amu that had seven carbons and included one carbonyl functionality, which was assigned to be 2-(3-oxobutan-2-yloxy)-2-hydroxypropanoic acid. The formation of these products derived from the ketyl and acetyl radicals is shown in Scheme 9. Acetoin was not detected in this reaction, and it was postulated that the organic products with masses 176 and 178 amu were thermally labile species, and the first one decomposed into acetoin as observed during the high temperature GC analysis in this work [5,82] and thus clarified its previous observation by Leermakers and Vesley [74]. Based on the evidence for acetyl and ketyl radical formation, the authors proposed a mechanism that proceeds through a proton-coupled electron transfer between the excited PA and a ground state PA that yielded acetyl and ketyl radicals [5,80,82]. The initial proton-coupled electron transfer step, shown in Scheme 9, is indistinguishable from H atom abstraction. However, none of the hydrogen atoms in PA are good candidates for H atom abstraction. Thus, a proton-coupled electron transfer, in which the electron and proton come from different sources (instead of together as a neutral H atom), was proposed.

**Figure 4:**
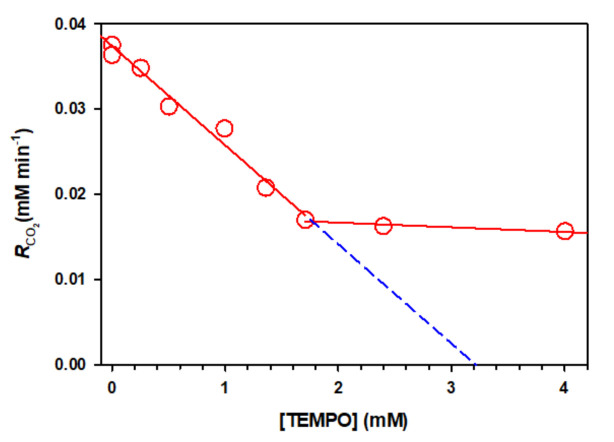
Rates of CO_2_(g) evolution ($R_{{\mathrm{CO}}_{2}}$) vs. the initial concentration of TEMPO for the 313 nm photolysis of fully deaerated 100 mM PA aqueous solutions at 293 K [5]. Copyright 2006, American Chemical Society.

Scheme 9 shows that the recombination of two ketyl radicals explained the formation of 2,3-dimethyltartaric acid. When the ketyl radicals combined with a ground state PA molecule and then an acetyl radical terminated the reaction, a β-ketocarboxylic acid intermediate was formed (not directly detected here), which then decarboxylated into the species with molecular weight 176 amu. The formation and subsequent decarboxylation of the β-ketocarboxylic acid to yield secondary CO_2_ also explained the results from experiments that the authors performed using radical scavengers and to monitor photodecarboxylation from the frozen state under variable temperature [82]. The work clearly distinguished that the secondary CO_2_ formed in the last reaction of Scheme 9 originated from a different source than the primary CO_2_ from decarboxylation of the acyloxyl radical in Scheme 8 [82]. Figure 4 shows how the evolution of CO_2_ from the aqueous photolysis could be only partially inhibited by the radical scavenger 2,2,6,6-Tetramethylpiperidinyloxy (TEMPO) as described by the linear fittings in red. The primary source of CO_2_ was so fast and efficiently produced that it could not be stopped even by 4 mM TEMPO. Instead, the secondary source of CO_2_ could be fully hindered by interrupting the free radical driven process starting at 1.7 mM TEMPO [5]. The blue dashed trace in Figure 4 shows the extrapolation indicating that the rate of CO_2_ evolution should have dropped to zero for 3.2 mM TEMPO if free radicals were exclusively controlling the generation of CO_2_ [5]. Finally, Guzman et al. demonstrated the hyperbolic dependence of the reaction on the initial concentration of PA which supported the multistep reaction mechanism presented [5].

**Scheme 10:**
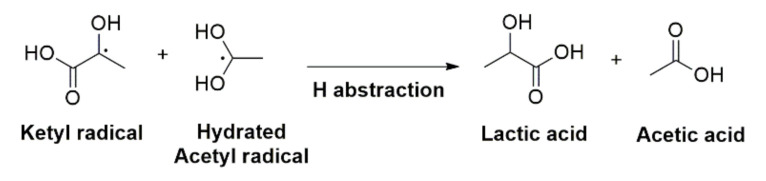
Proposed formation of lactic acid and acetic acid during PA photolysis in water from Griffith et al. [4].

The extensive work above on the mechanism by Guzman et al. served as an inspiration for new studies on aqueous PA photolysis. Recently, Griffith et al. proposed again that acetoin was a product along with lactic acid, acetic acid, and the carboxylic acid products [4] with molecular weight 176 and 178 amu identified by Guzman et al. [5] in the aqueous photolysis under atmospheric conditions. The suggestion for acetoin product was based on the gas phase FTIR spectrum of the headspace collected over the reaction and two-dimensional (2D) NMR of the stored post-photolysis mixture [4]. The formation of acetoin was proposed to proceed via the same reaction indicated by Closs and Miller in Scheme 6, and the formation of acetic and lactic acids is shown in Scheme 10.

**Figure 5:**
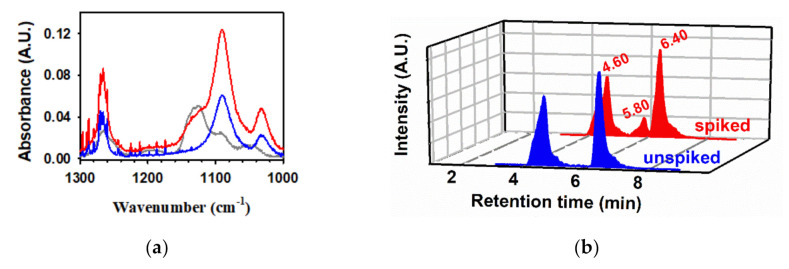

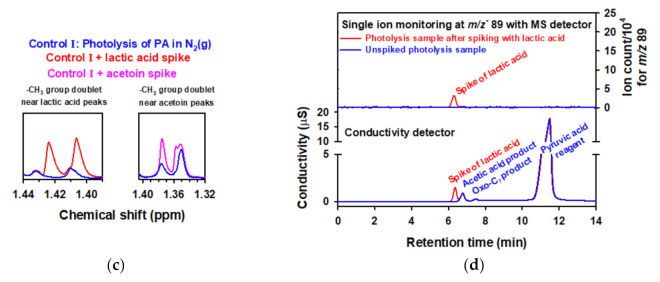
(**a**) Infrared spectrum of (blue line) gas products collected over 50 mM aqueous PA after photolysis (λ > 305 nm) for 1 h, (gray line) acetoin standard, and (red line) gas product spiked with acetoin clearly demonstrating the absence of this molecule in the headspace of the reactor [83]; (**b**) extracted ion (*m/z*^−^ = 267) chromatogram obtained by ultrahigh-pressure liquid chromatography/electrospray ionization(−)/MS (blue line) before and (red) after spiking the photolyzed PA sample for a final concentration of 20 μM acetoin prior to derivatization to form hydrazones. Retention times for the two hydrazones of PA are 4.60 and 6.40 min, and for the hydrazone of acetoin is 5.80 min (only observable in the spiked sample) but absent in the unspiked chromatogram [83]. (**c**) To the left hand-side is the ^1^H NMR spectrum for (blue), the −CH_3_ group doublet centered at ∼1.42 ppm in a control with 100.1 mM PA at pH 1.0 photolyzed at λ ≥ 305 nm for a 50% reagent conversion under continuous sparging with N_2_(g), and (red) the mismatch for this doublet after spiking to final concentration of lactic acid of 9.34 mM. To the right hand-side is the ^1^H NMR spectrum for (blue), the −CH_3_ group doublet centered at ∼1.36 ppm in the same control in left hand-side, and (pink) the mismatch for this doublet after spiking to an acetoin final concentration of 10.02 mM. This quality assurance analysis clearly demonstrated that lactic acid and acetoin are not photoproducts when working with well purified PA [77]. (**d**) At the bottom, the ion chromatogram is shown with conductivity detection for the region of elution of lactic acid for a control with 99.22 mM pyruvic acid at pH 1.0 photolyzed at λ ≥ 305 nm for a 21% reagent conversion under continuous sparging with N_2_(g) (blue) before and (red) after spiking to a final lactic acid concentration of 14.9 μM. The anions of all species were assigned with available standards and/or using the m/z^−^ values obtained by tandem mass spectrometry. At the top, the extracted ion chromatogram for lactic acid is shown (m/z^−^ 89) (blue) before and (red) after spike addition of lactic acid for the same samples in the bottom part of this panel, which completely discard the photoproduction of lactic acid when working with well purified PA [77]. Panels a and b: Copyright 2013, Proceedings of the National Academy of Sciences of the United States of America. Panels c and d: Copyright 2017, American Chemical Society.

Unfortunately, the FTIR spectrum in the study by Griffith et al. was complicated by water vapor that overlapped the signals of interest, making it truly impossible to confirm if the new products were really there. Thus, the analytical challenge was reapproached by Eugene et al., who carefully examined the headspace FTIR and liquid NMR spectral signals before and after spiking acetoin, lactic acid, and acetic acid *external standards* into the post-photolysis mixtures to confirm and/or discard the presence of these components in the mixture [2,77,83]. The careful quality assurance approach of Eugene et al. summarized in Figure 5 also included more conclusive chromatographic methods using multiple detectors and the use of derivatization techniques, which altogether served to reject the hypothesis of the photochemical formation of acetoin and lactic acid [2,77,83]. The absence of acetoin and lactic acid in the photoproduct mixture was demonstrated by comparing the results before and after spiking with standards (Figure 5) by the mismatch in (1) the FTIR spectra of gases, (2) chromatograms with multiple detectors and lower limits of detections and other spectroscopies, and (3) the ^1^H NMR spectra (chemical shift position, line shape, appearance of new peaks or shoulders, and intensity ratio for the −CH_3_ doublets) [2,77,83]. Instead, it became clear that the signals attributed to acetoin and lactic acid were contributed by the seven-carbon species (oxo-C_7_ product) of mass 176 amu identified by Guzman et al. [5], with possible contributions of its precursor β-ketocarboxylic acid intermediate shown in Scheme 9 [2,77,83]. In addition, low levels of acetic acid became evident in these analyses.

**Figure 6:**
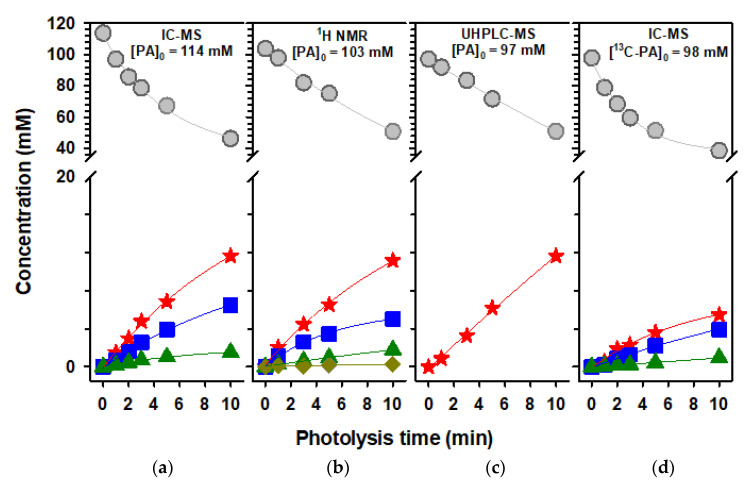
Quantitative time series for the aqueous photolysis at λ ≥ 305 nm of (gray circle) PA with the formation of (red star) the oxo-C7 product, (blue square) DMTA, (green triangle) the oxo-C8 product, and (gold diamond) acetic acid. The analytical method and initial concentration of PA are summarized at the top of each panel. (**a**) Ion chromatography (IC) with conductivity and mass spectrometry detection of anions. (**b**) Quantitative ^1^H-NMR. (**c**) UHPLC-MS from DNPH hydrazones of PA and the oxo-C_7_ product. (**d**) IC conductivity MS when using fully labeled ^13^C-PA. Copyright 2017, American Chemical Society [2].

The work of Eugene et al. also quantified the organic reaction products DMTA, the oxo-C7 product, the oxo-C8 product shown in Scheme 9 (the newly detected unstable β-ketocarboxylic acid intermediate), and acetic acid by multiple analytical methods as displayed in Figure 6 for IC conductivity MS, quantitative ^1^H NMR, and UHPLC-UV-MS of derivatized carbonyls to form hydrazones [2]. Figure 6 also displays the quantification of these products when using isotopically labelled ^13^C PA. Additionally, kinetic isotope effects in D_2_O as solvent provided strong support in favor of a proton-coupled electron transfer mechanism of photoreaction [2]. Future researchers interested in this work should be aware that, despite the results summarized above and in Figure 5 [2,77,83], the inaccurate suggestion that acetoin and lactic acid (likely from the use of impure reactant) could be photoproducts of PA photochemistry at λ ≥ 290 nm remains in the literature [7,84,85]. Moreover, most recently such confusion was expanded to provide an alternative structure and formation mechanism for a different oxo-C7 product with mass 176 amu [85], in a formation pathway (for a photoproduct that is quite major) that has been clarified to depend on a contaminant widely abundant in impure (not freshly distilled under vacuum) PA [77]. The unlikelihood of this alternative pathway depending on an impurity was discussed in detail in the follow up work of Eugene and Guzman along with an in-depth investigation of the sensitivity of the photoreaction to pH and oxygen content [86,87].

### 2.5. Heterogeneous Contributions to the Aqeuous Photochemistry of Pyruvic Acid

The surface acid-base behavior of this small 2-oxocarboxylic acid at the air–water interface has attracted recent attention, and it has been contrasted to acetic acid as a reference [88]. The results explained how PA molecules in the gas phase behave as they approach an aqueous surface such as those of a deliquesced atmospheric aerosol or a fog droplet [88]. The experimental work employing surface sensitive online electrospray ionization mass spectrometry (OESI-MS) showed that, upon titration of gaseous PA and acetic acid molecules impinging on the surface of aqueous microdroplets, the inflection points corresponding to the p*K_a_* value of PA were positioned at 1.8 pH units lower than in the bulk phase [88]. This shifting was attributed primarily to the increased proton affinity of the interface, while the difference between PA and acetic acid was explained as a consequence of the different hydration configuration of the acid molecules on the surface of water [88]. It is known that four water molecules actively participate in the cooperative hydration of the carbonyl group of PA into its monohydrate (2,2-dihydroxypropanoic acid), even in ice down to 230 K [22]. Furthermore, four water molecules are key in this equilibrium as they are able to reproduce the solvation of PA, and be used to model its UV spectrum with a small cluster [89].

**Figure 7:**
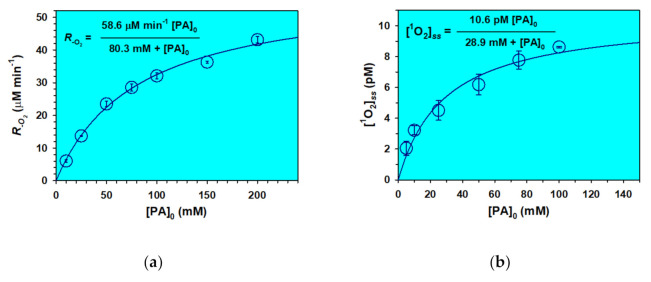
(**a**) Rate of dissolved oxygen decay ($R_{O_{2}\left(aq\right)}$) at variable initial concentration of PA during λ ≥ 305 nm irradiation of solutions initially saturated with air. The complete experimental conditions can be found in the original work [86]. Adapted from data published in Eugene and Guzman; The Effects of Reactant Concentration and Air Flow Rate in the Consumption of Dissolved O2 during the Photochemistry of Aqueous Pyruvic Acid. Molecules. 2019, 24 (6), 1124; (**b**) steady-state singlet oxygen production ([^1^O_2_]*_ss_*) during 1 h irradiation at λ ≥ 305 nm of under continuous and stable saturation with the air of PA solutions. Other experimental conditions can be found in the original work [87]. Copyright 2019, American Chemical Society.

Recent PA photochemistry results also demonstrated the importance of the mass transfer of O_2_(g) into water for producing carbon centered radicals with a Langmuir dependance (Figure 7a) [86]; the ability of photoexcited PA to form singlet oxygen (Figure 7b), a contributor to atmospheric oxidations in aerosols; and the importance of closing the mass balance between the reactant and products during the time series as encapsulated by the comparison of their quantum yields [87]. Keeping in mind that the transfer of O_2_ from the gas phase into water is a thermodynamic and kinetic problem related to the interfacial boundary, it is possible to explain that the dissolved O_2_ decay rate, $R_{O_{2}\left(aq\right)}$, during the photochemistry of PA initially saturated with air displays a Langmuir isotherm type of dependence on the organic acid concentration. This Langmuir dependence is presented together with its hyperbolic fitting in Figure 7a. Not only do the intermediate carbon centered radicals (R^•^) consume dissolved oxygen, resulting in peroxyl radicals (RO_2_^•^) of atmospheric importance, but the triplet excited state of PA also creates a steady state population of singlet oxygen ^1^O_2_* with the dependance on PA concentration presented in Figure 7b, which reaches a plateau at 10.6 pM ^1^O_2_ [87]. In addition, experiments using liquid films of PA photolyzed in the presence of a 100 ppb ozone exhibited dominant surface chemistry over that registered in the bulk, pointing out the importance of this heterogeneous process [90]. Similarly, other attempts have aimed to investigate the *m/z* value of possible oligomers in heterogeneous chemistry [91,92], although regularly battling with the presence of impurities before starting the reactions.

### 2.6. Ionic Strength Effect, Optical Properties, and Cross Photoreaction in Water

**Scheme 11:**
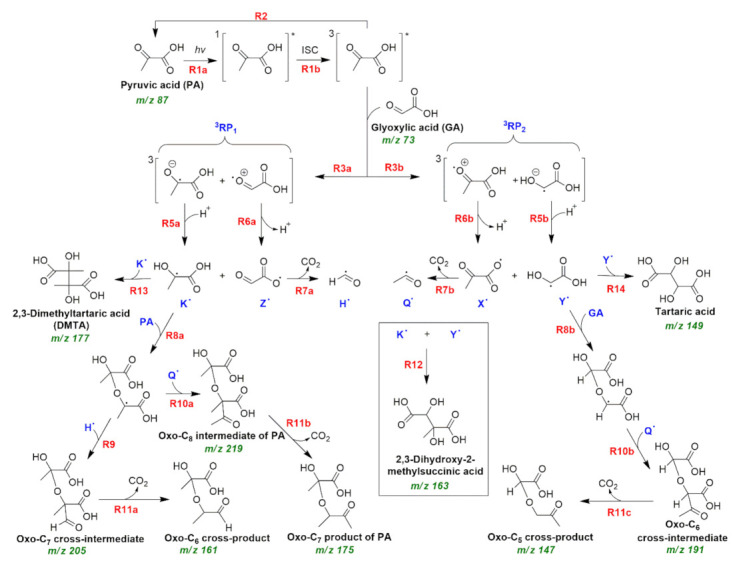
Detailed mechanism for the cross photooxidation of PA and GA at λ ≥ 305 nm in water, reproduced with permission from ref. [98]. Copyright 2018, American Chemical Society.

The use of an innovative experimental strategy demonstrated that the oligomers generated in the photolysis of PA in water form supramolecular interactions and undergo a cyclic enhanced red-shifted visible absorption in the dark, which is enhanced by dissolved electrolytes increasing the ionic strength [93], while photobleaching occurs under sunlight [94]. Other approaches pursued the direct assignment of oligomer structures based on their *m/z* value in complex mixtures of reaction products with varied polarity [95]. Importantly, the exploration of the ionic strength effect with a focus on the absorption spectrum of PA supported an increase in photolysis rate, absorption intensity, and red-shift for higher concentrations of electrolytes [96]. In addition, the effects of Na^+^ and Ca^2+^ to deprotonate PA increasing the carbonyl form in hydration equilibrium were enhanced for the dication but resulted in a drop of the photolysis rate [97]. Furthermore, the detailed mechanism for the cross photoreaction of PA and GA in water with electrolytes under high ionic strength and without electrolytes was reported [98]. Once excited, PA and GA initiate proton-coupled electron transfer for the former or hydrogen abstraction and α-cleavage reactions for the latter [98]. The mixture of cross photoproducts, including trimers, exhibited a time-domain periodicity of the optical properties due to the chromophores that undergo alternating thermochromism and photobleaching between nighttime and daytime cycles, respectively, as depicted in Scheme 11 [98].

Reproducible photobleaching and thermochromism cycles were registered when alternating irradiation and thermal aging stages [98]. An O_2_(g)-saturated atmosphere had a detrimental effect on the development of thermochromism, with an increase in absorption of products favored under anoxic conditions. The cross photoreactivity of PA and GA resulted in species with higher O/C ratios than for the PA self-reaction [98]. While GA also increased the reactivity channels of PA, the latter also behaved as a photosensitizer that induced greater reactivity for GA as an example of other complex precursors available in the atmosphere [98]. The work also served as an example for how other organic constituents present in atmospheric waters can compete for UV light.

**Scheme 12:**
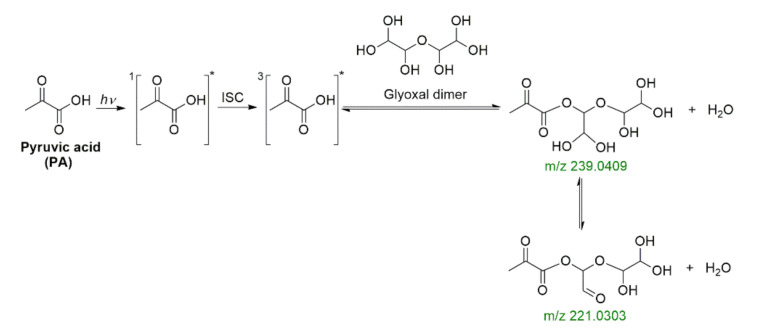
Proposed reaction pathway for the Fischer esterification of a glyoxal dimer with the triplet excited state of PA, which was described in ref. [99].

A recent study applied ultrahigh resolution mass spectrometry to provide molecular-level understanding of the photosensitized reaction of PA with glyoxal (GL) based on the assignment of elemental formulas [99]. The work was supplemented by ab-initio calculations that supported the cross-reaction PA + GL rather than the self-reaction of PA based on the abundant highly oxygenated multifunctional compounds proposed as products [99]. The cross-products from the PA + GL were described to mainly form upon Fischer’s esterification. For example, the nucleophilic -OH group of a dimer of GL reacts with the highly reactive -COOH group of the triplet excited state of PA as depicted in Scheme 12, which implies a dehydration should have proceeded in the aqueous media [99].

### 2.7. Future Directions for Aqueous Phase Photoreactions Studies in Atmosphere

We provided a brief description of some representative photochemical studies with small 2-oxocarboxylic acids above, where water played a key role in the observed chemistry. Indeed, aqueous tropospheric chemistry can be influenced by photochemical reactions, which provide reactive species, an emerging issue that deserves more attention. Despite the substantial progress that has been made in this field, the current comprehension of water solvation effects in the photochemistry is still in progress. Future laboratory experiments, computational quantum mechanical modeling, and molecular dynamic simulations could advance our understanding of effects such as solvation, stabilization, and solute position and orientation [88,100] in the photoprocesses in bulk and interface chemistry. Because the photochemical products of these reactions can contribute to the reservoir of light absorbing species in atmospheric waters, they can also play an active role in the aging of aqueous SOA, i.e., by triggering photosensitized reactions. Thus, quantitative understanding of the radicals and reactive oxygen species (ROS) produced from the photochemistry of 2-oxocarboxylic acids and of their implications in multiphase ageing of particulates is vital, as should also be the comparison with other competitive loss pathways. Studying how the generated excited triplet states directly and indirectly alter the organic surface-active components typically coating the air–water interface of atmospheric particles is of major importance. Addressing such problems should inform us of new processes that can modify the morphology and properties of atmospheric particles and their influence on climate forcing processes [101].

## 3. Method

This work originated from a systematic literature search aimed to offer a narrative synthesis performed following the recommendations from the Preferred Reporting Items for Systematic Reviews and Meta-analysis (PRISMA) statement [102]. Two hundred and ninety-nine papers appearing in the international database Web of Science from Clarivate Analytics were identified by the following combinations of keywords: (1) pyruvic acid or 2-oxopropaoic acid, or (2) glyoxylic acid or 2-oxoethanoic acid, (3) photo*, and (4) water or gas. The use of the symbol “*”as a wildcard (or truncation) facilitated the broad identification of common terms starting with “photo”such as photolysis, photochemistry, photodissociation, photoinduced, etc. The search was constrained to articles published until 15 August 2021. For the inclusion criteria, papers (research articles, review articles, proceedings papers, and early access) published in journals that provide physical and theoretical insights mainly in the fields of physical, organic, and analytical chemistry and environmental and related sciences were given preference, only if dealing with the small 2-oxocarboxylic acids of interest. Instead, the exclusion criteria applied to remove 28 papers with unnecessary content considered to be out of the scope for this review were: (i) studies with C4 or longer chain 2-oxocarboxylic acids, (ii) studies dedicated to molecular biology, nanoscience and nanotechnology, plant sciences, applied physics, biotechnology, agronomy, medical and biophysical fields, and (iii) astrobiology related research. A careful revision of the abstracts for verifying their relevance to this review enabled the exclusion of 97 records. After analyzing the methodology and results of the remaining 174 papers, we excluded 74 reports that did not meet our inclusion criteria. Thus, 100 individual reports were included in our first version of the work with any needed additional support references. Finally, six additional references emerging from peer-reviewing were included for a total of 106 reports.

## 4. Conclusions and Atmospheric Implications

The formation of SOA in atmospheric waters currently underpredicted in atmospheric models, likely due to previous limitations to identify the sources such as the photochemical processing of small 2-oxocarboxylic acids. Consequently, it has been difficult to constrain the uncertainties of estimates of atmospheric aerosol loading and hence the effect that organic aerosols have on the energy balance of Earth’s climate. Importantly, it was shown that aqueous GA irradiation under atmospheric conditions was able to produce glyoxal [3], a reactive dicarbonyl species responsible for the production of as much as 14% of summertime SOA over the southeast United States alone [103]. This corresponds to an additional 0.8 μg m^−^^3^ of SOA contributed to the local atmosphere from glyoxal. The UV-visible and fluorescence study of glyoxylic acid photoproducts indicated that irradiation-dark aging cycles that imitate day/night changes allowed for destruction and then regeneration of solar actinic absorbing compounds [3], a behavior also observed for PA and for the cross photoreaction of PA and GA [2,93,94,98]. Atmospheric aerosols made up of small 2-oxocarboxylic acids or containing such a terminal group in their structure will imprint large fluctuations in the optical properties, and affect the total radiative forcing of organic aerosols (RFari in Figure 2). The presence of electrolytes and air reduces the thermochromism recovered during dark aging [3]. The overall trend across alternating daytime and nighttime processing stages of GA showed a shrinking absorption profile with increasing number of diurnal cycles, an indication that these types of aerosols should absorb less while they age, contributing a net cooling effect. The quantum yield of GA photodecomposition (Φ_-GA_ = 1.78% for pH 1, with background electrolytes: [Na^+^] = 468 mM; [Cl^−^] = 545 mM; [SO_4_^2^^−^] = 28.2 mM under air saturated conditions) [3] can now be implemented in atmospheric models, and help to improve current estimates of atmospheric SOA loadings.

A comparison of the importance of direct photolysis of GA vs. indirect oxidation by HO^•^ indicates that direct photolysis is, while not the primary fate, important enough to be included in models [3]. The lifetimes of GA against the two possible aqueous phase processes are 11 days for direct photolysis vs. 5 days for reaction with HO^•^ radical. For these, two dominant aqueous phase processes experienced by GA, then about 4.99 Tg GA per year [44] are lost to direct photolysis, yielding glyoxal, formic acid, oxalic acid, tartaric acid, CO, and CO_2_ [3]. The molar yield of glyoxal at early reaction times is about 40% of the GA consumed, which represents the lower limit of glyoxal that can be produced from GA under these conditions because it is at steady state. Therefore, an estimated 1.99 Tg GA can be converted to glyoxal through aqueous phase processing each year, yielding at least 1.56 Tg of glyoxal per year or 3.5% of the total 45 Tg estimated to be emitted per year [104]. A recent study on SOA formation from glyoxal determined that about 5 μg m^−^^3^ SOA could be generated by the processing of glyoxal at a level of 50 mM in the aqueous phase [105], indicating that the glyoxal generated from GA photolysis ([glyoxal]_ss_ = 2 mM) may contribute more than 0.2 μg m^−^^3^ to the global yearly SOA budget, equivalent to 3.5% of the summertime SOA observed over the southeastern United States [103].

The quantum yield measured for PA [2] verified that direct photolysis was the dominant aqueous fate, and that aqueous photochemistry can compete significantly with gas phase photolysis because of the ease with which gaseous pyruvic acid partitions into atmospheric water. The large partitioning was in part explained by the enhanced acidity of this carboxylic acid at the air–water interface of aerosols [88]. This enhanced acidity described a shift in equilibrium that provides more of the highly water-soluble carboxylate anion to the interface, increasing the likelihood of carboxylic acids dissolving into the bulk phase of atmospheric water to undergo subsequent chemical reactions. Given the relative lifetimes of PA toward direct photolysis and HO^•^ radical oxidation in both the gas and aqueous phases [2], direct aqueous photolysis is likely responsible for at least 34% (based on the rate constants for each process) of the PA destroyed in the atmosphere each year or 0.292 Tg or of PA per year, second only to gas phase photolysis. Furthermore, the quantum yields measured steady state dissolved oxygen levels showed that each PA molecule lost contributes to an oxidized organic product that will remain in the particle phase to contribute to SOA formation [87]. Using the relative product yields of the direct photolysis mechanism with the quantum yields indicated in the literature [87], the total SOA burden of direct aqueous photolysis from PA is estimated to be 0.257 Tg per year. While this SOA production was less than that expected from GA, mostly due to the lower amounts of PA found in the atmosphere, the high reactivity of the radicals photogenerated from PA toward dissolved oxygen and, ostensibly, other organic compounds that could be present in atmospheric aerosols suggested that PA may act as an intermediate or promotor or sensitizer in various atmospheric chemical cycles. PA photolysis was also found to be a source of singlet oxygen missing from current models, with a singlet oxygen production that is on par with that of irradiated natural organic matter extracted from fog water [87,106]. Overall, the work in this review has contributed to creating a better understanding of aqueous SOA formation pathways from small 2-oxocarboxylic acids, molecules that can also assist future studies with comparing other SOA precursor molecules in aerosol water.

## Data Availability

Not applicable as no new data were created in this review.
